# The Association Between Fluid Overload and Endothelial Dysfunction in Chronic Kidney Failure Patients Undergoing Hemodialysis Twice a Week

**DOI:** 10.7759/cureus.44381

**Published:** 2023-08-30

**Authors:** Adi Wijaya, Maruhum Bonar Hasiholan Marbun, Pringgodigdo Nugroho, Ikhwan Rinaldi

**Affiliations:** 1 Internal Medicine, Cipto Mangunkusumo Hospital - Faculty of Medicine Universitas Indonesia, Jakarta, IDN

**Keywords:** hemodialysis, hyperhydration, endothelial dysfunction, brain natriuretic peptide, asymmetrical dimethylarginine

## Abstract

Background: Fluid overload causes excessive systemic vasoconstriction and decreased perfusion of peripheral tissues, leading to abnormalities in cardiopulmonary physiological functions. Prolonged fluid overload caused by inadequate hemodialysis may cause heart dilatation, left ventricular hypertrophy, hypertension, and a decrease in coronary reserves, which later will develop into coronary ischemia, leading to increased morbidity and mortality of cardiovascular disease (CVD). Endothelial dysfunction plays a role in excessive vasoconstriction on fluid overload. Brain natriuretic peptide (BNP) and asymmetric dimethylarginine (ADMA) are used as parameters of fluid overload and endothelial dysfunction, respectively. This study is conducted to describe the relationship between fluid overload with endothelial dysfunction.

Method: This study is a cross-sectional study of kidney failure patients who underwent hemodialysis twice weekly for at least three months. BNP and ADMA were used as parameters for fluid overload and taken prior to hemodialysis.

Result: From 126 subjects, the proportion with fluid overload (BNP>356 pg/ml) was found to be 64.3% with the median age of subjects being 52 years (47-62). There was 47.6% population with endothelial dysfunction (ADMA>100 ng/ml). Presumptive causes of primary chronic kidney disease (CKD) were hypertension (38.9%), diabetes mellitus (DM) (28.6%), and glomerulonephritis (21.4%). There was no significant association between fluid overload and endothelial dysfunction (PR=1,042, p=0.832 CI 95%=0.714-1.521).

Conclusion: There was no relationship between fluid overload and endothelial dysfunction.

## Introduction

The need for renal replacement therapy (RRT) in Indonesia increases as the incidence of chronic kidney disease (CKD) rises annually. Treatment modalities for end-stage kidney disease (ESKD) that are currently available in Indonesia include hemodialysis, continuous ambulatory peritoneal dialysis, and kidney transplant. Chronic hemodialysis procedure is usually performed in CKD patients and has become one of the most common RRTs in Indonesia. In the 10th Indonesian Renal Registry (IRR) annual report, in 2017, there were 30,831 new patients who underwent hemodialysis as compared to 4,977 new patients in 2007 [1]. ESKD was associated with 20 times higher cardiovascular disease (CVD)-related mortality as compared to the normal population. Around 80% of hemodialyzed patients had left ventricular abnormalities such as left ventricle dysfunction prior to the initiation of hemodialysis, which can be predictors of ischemic heart disease and heart failure [2]. The rate of mortality in these patients reached 18% annually and half of it is associated with CVD [3]. The five-year mortality rate of hemodialysis in patients with and without a history of CVD is 53% and 24%, respectively [4].

One of the main purposes of chronic hemodialysis in ESKD is to obtain normohydration status [5]. Inadequate frequency of hemodialysis will lead to fluid overload, causing an increase in morbidity and mortality [5-7]. Fluid overload will cause activation of the renin-angiotensin-aldosterone system, neurohormonal, and renal blood vessels. This will cause vasoconstriction, ventricular hypertrophy, and cardiac dilatation, leading to decreased coronary reserve, causing oxidative stress and inflammation, and resulting in an inadequate balance between nitric oxide (NO) and reactive oxygen species (ROS). The imbalance decreases NO bioavailability and increases ROS along with asymmetric dimethylarginine (ADMA), causing endothelial dysfunction, exacerbating existing vasoconstriction, and increasing myocardial damage. In addition, endothelial dysfunction is also influenced by several factors such as hypertension, hyperlipidemia, diabetes, smoking, age, and hyperhomocysteinemia [8]. Fluid overload will extend the length of stay in the hospital and is an important predictor of the outcome of ESKD patients with dialysis-related complications of CVD [9]. The frequency of hemodialysis twice a week, which is mostly done in Indonesia, has a higher potential for interdialytic fluid overload compared to patients who undergo hemodialysis more frequently than what is recommended by the Kidney Disease Outcomes Quality Initiative [1]. Yussac et al. stated fluid overload occurred in 60% of patients with hemodialysis two times a week [10]. In line with this study, Arsana et al. also stated that fluid overload occurred in 62% of patients undergoing hemodialysis twice a week, measured using brain natriuretic peptide (BNP) as a fluid overload parameter [11].

The gold standard for measuring hydration status can be carried out by means of dilution of radioisotopes of iodine or chromium; however, these methods are very expensive and difficult to practically apply in clinical settings [12]. Hence, BNP is proposed to be a parameter that can be applied in clinical settings. Besides its ability to assess fluid overload conditions in hemodialysis patients, BNP biomarkers can also be used to predict mortality. Plasma BNP and NT-proBNP levels in chronic hemodialysis patients are highly sensitive and specific in describing fluid overload conditions, increased ventricular wall pressure due to left ventricular hypertrophy, and left ventricular systolic dysfunction, as well as predictors of CVD-related mortality in chronic hemodialysis patients [13]. Plasma BNP levels as a screening for pre-hemodialysis fluid overload conditions have a sensitivity of 94% and a specificity of 30% [14], but the cut-off point value as a determinant of fluid overload is found to be diverse across studies [13].

Currently, there has been no study on the relationship between fluid overload and endothelial dysfunction in twice-weekly hemodialysis patients using BNP levels as a biomarker of fluid overload and ADMA as a biomarker of endothelial dysfunction. Further studies are important to evaluate this relationship in order to reduce morbidity and mortality that can be caused by hemodialysis-related fluid overload and improve the quality of hemodialysis modalities in Indonesia. This study is conducted to describe the relationship between fluid overload and endothelial dysfunction in twice-weekly hemodialysis patients. This is a full study from a submitted abstract in Kidney International Reports (https://doi.org/10.1016/j.ekir.2022.01.696).

## Materials and methods

This cross-sectional study was conducted in September 2020-2021 at the Hemodialysis Unit of Cipto Mangunkusumo National Referral Hospital, Jakarta. Inclusion criteria were stable CKD patients who had undergone hemodialysis twice weekly for at least three months, 18 years old or older. Exclusion criteria were patients with severe heart failure (NYHA III-IV), malignancy, pregnancy, liver cirrhosis, other critical conditions, and taking corticosteroids and immunosuppressants. The informed consent form, which was written in Bahasa Indonesia, is shown in the Appendices section (Supplementary 1). The study has been granted ethical clearance (KET-888/UN2.F1/ETIK/PPM.00.02/2020) by the Ethical Committee of the Faculty of Medicine, Universitas Indonesia.

Arsana et al. described the proportion of high BNP plasma in patients undergoing chronic hemodialysis twice weekly as 62% [11]. Using this data, the estimated sample size for this study was measured and the minimum sample required is 124. The formula was described in the Appendices section (Supplementary 2). Informed consent was obtained prior to data collection. Subject characteristics including age, sex, and pre-existing conditions related to CKD (glomerulonephritis, diabetes mellitus (DM), hypertension, and unspecified) were collected by the physician using history taking and medical records. Subjects also underwent physical examinations and blood sample collection. Samples were taken shortly before hemodialysis. The estimated sample size was 94, and the formula was described in the Appendices section (Supplementary 3). ADMA and plasma BNP samples from eligible subjects were collected through a blood sample and further analyzed, where the ADMA sample was analyzed using liquid chromatography-tandem mass spectrometry (LC-MS) and the plasma BNP sample was analyzed using enzyme-linked immunosorbent assay (ELISA).

The research data was recorded on the research form and was coded. Data was analyzed using SPSS Statistics version 22 (IBM Corp. Released 2013. IBM SPSS Statistics for Windows, Version 22.0. Armonk, NY: IBM Corp.) and then presented in the form of a percentage (%), mean ± SB, or median. Analysis of the relationship between fluid overload and endothelial dysfunction was conducted using chi-square analysis. Multivariate analysis was used to identify confounding factors such as fluid overload, age, blood pressure, obesity, smoking, DM, inflammation, medications, and dyslipidemia. Logistic regression analysis was performed to see the relationship between fluid overload and endothelial dysfunction and other influencing factors. The p-value <0.0550 was used to determine the level of statistical significance.

## Results

**Figure 1:**
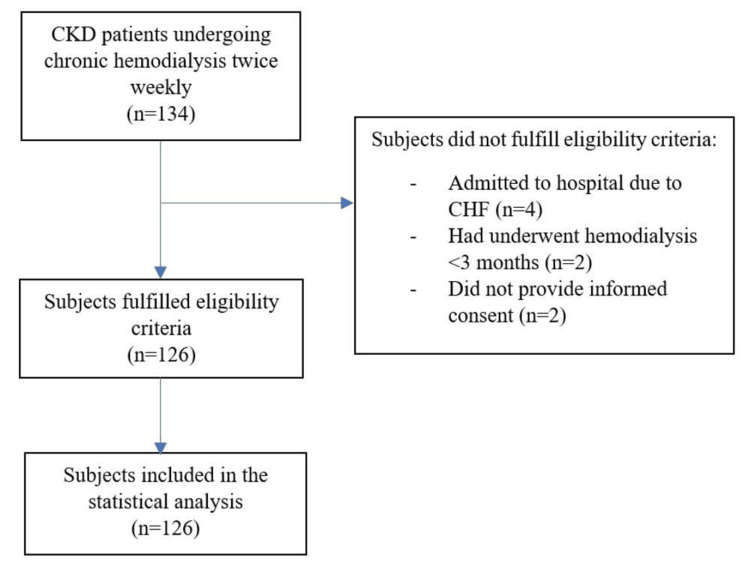
Flow chart of subject recruitment CKD: chronic kidney disease, CHF: congestive heart failure

A total of 134 patients in Cipto Mangunkusumo National Referral Hospital underwent chronic hemodialysis procedures from September 2020 to September 2021. Eight patients were excluded, and a total of 126 subjects who fulfilled the eligibility criteria were included in the study. The patient recruitment process was further elaborated in Figure 1.

Table 1 describes the characteristics of the research subjects. The median age was 52 years (47-62), with 80.2% of the subjects aged less than 65 years. Fifty-four percent of the research subjects were women. Presumptive causes of CKD were hypertension (38.9%), type 2 DM (28.6%), and glomerulonephritis (21.4%). The median BNP level in 126 subjects was 663.75 pg/ml (281.93-1666.28). Meanwhile, there were 64.3% of subjects who had BNP levels >356 pg/ml. The average ADMA level in all subjects was 99.17 ± 29.61ng/ml, yet there were 47.6% of subjects who had ADMA levels >100 ng/ml.

**Table 1:** Characteristics of research subjects ^a^Median ^b^Mean ADMA: asymmetric dimethylarginine, BMI: body mass index, BNP: brain natriuretic peptide, CDL: catheter double lumen, CRF: chronic renal failure, HsCRP: high-sensitivity C-reactive protein, DM: diabetes mellitus

Variable	∑Subject	Percentage	Mean/median
Age			52(47-62)^a^
<65 years	101	80.2	
≥65 years	25	19.8	
Sex			
Female	68	54	
Male	58	46	
Working outside			
No	80	63.5	
Yes	46	36.5	
Smoking			
No	101	80.2	
Yes	25	19.8	
Diuresis >200 ml per day			
No	103	81.7	
Yes	23	18.3	
Presumptive causes of CRF			
Kidney agenesis	2	1.6	
Diabetic kidney disease	36	28.6	
Hypertension	49	38.9	
DM + hypertension	4	3.2	
Glomerulonephritis	27	21.4	
Obstruction-infection	2	1.6	
Polycystic kidney disease	3	2.4	
Unknown	3	2.4	
DM			
No	89	70.6	
Yes	37	29.4	
Hypertension			
No	23	18.3	
Yes	103	81.7	
Number of hypertension drugs			
≤1	67	53.2	
>1	59	46.8	
Hepatitis C			
No	105	83.3	
Yes	21	16.7	
BMI category			
Non-obesity	68	54	
Obesity	58	46	
Vascular access			
Non-CDL	47.6	60	
CDL	52.4	66	
BNP			663.75 (281.93-1666.28)^a^
High (>356) pg/ml	64.3	81	
Low (≤356) pg/ml	35.7	45	
ADMA			99.17±29.61^b^
Levels >100 ng/ml	47.6	60	
Level ≤100 ng/ml	52.4	66	
HsCRP			4.65 (1.78-13.43)^a^
Content >3	57.9	73	
Level ≤3	42.1	53	
Albumin			
Levels ≥3.5 g/dl	97.6	123	
Levels <3.5 g/dl	2.4	3	

Table 2 describes the relationship between fluid overload and endothelial dysfunction in patients with ESKD undergoing hemodialysis twice weekly. The chi-square test found no relationship between fluid overload and endothelial dysfunction (p=0.832; 95% CI 0.714-1.512).

**Table 2:** The relationship between fluid overload and endothelial dysfunction in patients with end-stage renal disease undergoing hemodialysis twice a week BNP: brain natriuretic peptide, ADMA: asymmetric dimethylarginine

Variable	ADMA levels (endothelial dysfunction status)				PR (95% CI)	p-value
	Yes (≥100 ng/mL)		No (<100 ng/mL)			
	∑	%	∑	%		
Hydration status (BNP level)						
Yes (>356 pg/ml)	38	46.9	43	53.1	1.042 (0.714-1.521)	0.832
No (≤356 pg/ml)	22	48.9	23	51.1		

Table 3 describes the relationship between confounding factors (fasting blood glucose control, smoking, inflammation, age, obesity, and hypertension medication) and endothelial dysfunction using ADMA. The chi-square test showed that inflammation was a factor strongly associated with endothelial dysfunction (PR 6.769 (2.917-15.710); p<0.001).

**Table 3:** Relationship between confounding variables (fasting blood sugar, smoking, age, inflammation, obesity, hypertension drugs) with endothelial dysfunction using ADMA HsCRP: high-sensitivity C-reactive protein

Variable	Endothelial dysfunction		PR (95% CI)	p-value
	Tall (≥100 ng/mL)	Low (<100 ng/mL)		
Fasting glucose level (>126 g/dL)				
Yes	14 (42.4)	19 (57.6)	0.805 (0.512-1.265)	0.440
No	39 (52.7)	35 (47.3)		
Smoke				
Yes	12 (48.0)	13 (52.0)	1.010 (0.640-1.594)	1,000
No	48 (47.5)	53 (52.5)		
Age				
≥65 years	49 (48.5)	52 (51.5)	1.103 (0.678-1.792)	0.856
<65 years	11 (44.0)	14 (56.0)		
Inflammation (HsCRP)				
High (>3 mg/l)	55 (70.5)	23 (29.5)	6,769 (2,917-15,710)	<0.001
Low (<3 mg/l)	5 (10,4)	43 (89.6)		
Obesity				
Yes	18 (51.4)	17 (48.6)	1.114 (0.754-1.647)	0.740
No	42 (46.2)	49 (53.8)		
Antihypertensive drugs				
Yes	43 (45.3)	52 (54.7)	0.825 (0.560-1.217)	0.472
No	17 (54.8)	14 (45.2)		

Multivariate analysis of hyperhydration and confounding factors showed a >10% OR difference between hyperhydration and confounding factors when associated with endothelial dysfunction. Even so, the addition of confounding factors to hyperhydration still did not show a statistically significant relationship between hyperhydration and endothelial dysfunction. The data are described in Table 4.

**Table 4:** Multivariate analysis and logistic regression HsCRP: high-sensitivity C-reactive protein

Variable	PR (95% CI)	p-value	Delta OR
Crude			
Hyperhydration	0.748 (0.328-1.704)	0.489	
Adjusted			
+ HsCRP	1.604 (0.551-4.666)	0.386	53.37%
+ Fasting glucose level	1.614 (0.551-4.728)	0.383	0.62%
+ Antihypertensive drugs	1.628 (0.553-4.790)	0.376	0.85%
+ Obesity	1.622 (0.550-4.778)	0.381	0.37%
+ Age	1.677 (0.562-5.009)	0.354	3.27%
+ Smoking	1.660 (0.545-5.053)	0.373	1.02%

## Discussion

The median age of the subjects between 41 and 62 years old in this study was found to be 52 years old, with 80.2% of the subjects <65 years old. This proportion is higher than the study by Lee et al. [15] which found the proportion of subjects aged <65 years old to be 65.9%. The proportion of subjects aged <65 years in this study is almost the same as the one in IRR data which stated 60% of the subjects with an age range of 45-64 years [1]. The median age of the subjects in this study was lower than the age of the subjects in the study by Chazot et al. [16] with a median age of 75.4 years. The median age of the subjects who were younger in this study is suspected to be related to the presumptive cause of most CKD due to glomerulonephritis, which was not recognized early; therefore, patients were already falling into end-stage renal disease. The causes of chronic renal failure (CRF) in the study by Chazot et al. [16] were predominantly due to metabolic and degenerative diseases. A higher median was found in a study by Chazot et al. [16] (mean 74.5 years) with the most common cause of CRF being DM (41%); likewise, in a study by Hwang et al. [17] where the mean age of the subjects was 61 years, the most common cause of CRF was DM (46%).

The proportion of women is higher than men (54% and 46%, respectively). This proportion is different from data on chronic hemodialysis patients in IRR, where males had a higher proportion as compared to women (56% and 44%, respectively) [1]. In addition, the study by Chazot et al. [16] found that the proportion of men was higher as compared to women (66% and 34%, respectively). From these studies, it could be inferred that there could be an association between sex and age in the CRF population who undergo hemodialysis.

The most presumptive causes of CRF in this study were glomerulonephritis (21.4%), DM (28.6%), and hypertension (38.9%). The Indonesian Society of Nephrology (PERNEFRI) in the IRR 2017 found that DM is the most presumptive cause of CRF followed by hypertension and glomerulonephritis [1]. Similarly, Chazot et al. [16] found that 41% of CRF cases were caused by DM. On the other hand, a study in Japan [18] described 43% of CRF caused by glomerulonephritis, 30% of which were caused by DM. This variability of data indicates that there are further causes of CRF that remain underdiagnosed. This variability is assumed to happen due to many CRF patients with previously undefined causes of glomerulonephritis, leading to underdiagnosed CRF.

Most of the comorbidities in this study were hypertension with a proportion of 81.7%, where 46.8% of them were patients who consumed two to four combinations of hypertension drugs. Besides that, other comorbidities identified from subjects are DM (29.4%) and chronic heart disease (17.5%). The high co-morbid hypertension and the difficulty of controlling blood pressure in chronic hemodialysis twice weekly are thought to be related to fluid overload [10,19]. The proportion of patients with DM comorbidity in the study by Lee et al. [15] was found to be higher, amounting to 46.9%. In chronic hemodialysis patients with comorbid DM, the average predialysis plasma BNP was 571.7 pg/ml, which was higher as compared to the average predialysis plasma BNP of 319.4 pg/ml in those without comorbid DM. A study by Igarashi et al. [20] on 223 subjects with type 2 DM found an increase in plasma BNP associated with age, comorbid hypertension, and comorbid congestive heart disease. However, this is independent of the duration of DM, HbA1c level, and hyperlipidemia. The exact mechanism of increased plasma BNP in DM patients is currently unknown. It is thought to be related to the induction of sodium retention and volume expansion, as well as the downregulation of NPR-A receptors in the renal tubules [20].

Predialysis systolic blood pressure in this study obtained an average of 137.75 (± 24.37) mmHg with a proportion of hypertension of 81.7%. A study by Jhee et al. [21] found that predialysis systolic blood pressure <110 mmHg and ≥170 mmHg in chronic hemodialysis patients increased the risk of death with an observation period of 4.5 years. Similarly, a study by Stidley et al. [22] found predialysis TDS <120 mmHg and ≥150 mmHg increased mortality in chronic hemodialysis patients. Predialysis systolic blood pressure ≥160 mmHg increases the risk of death related to CVD 2.19 times within five years in chronic hemodialysis patients three times weekly [23]. High predialysis systolic blood pressure in this study is thought to be associated with a high proportion of fluid overload and suboptimal blood pressure control.

The average ADMA level in this study was 99.17 (± 27.61) ng/ml. This finding is similar to the study conducted by Betlin et al. [24] of 115.14 (± 34.34) ng/ml and the study by Dogan et al. [25] of 111.1 (±60.6) ng/ml. However, Dogan et al. [25] also delivered that a significant difference in the ADMA level was found between the normovolemic groups of 82.82 (± 8.08) ng/ml and the hypervolemic group of 139.38 (±115.14) ng/ml. Although this study did not analyze in detail the differences in mean ADMA levels in the hyperhydrated group (BNP>356 pg/ml) and the normohydrated group (BNP≤ 356 pg/ml), it appears that an increase in BNP levels is not necessarily followed by an increase in ADMA levels. Zhang et al. [26] described that there was a decrease in ADMA levels in conventional hemodialysis patients who underwent three times weekly by 12.84 ± 6.69 µmol/week. In addition, Kielstein et al. [27] found that there was a decrease in ADMA levels of ± 65% at five hours after the hemodialysis process and ADMA levels would increase again at 18 hours after hemodialysis approaching the pre-hemodialysis value. These findings might explain the reason ADMA levels remained high in hemodialysis patients as increased ADMA level is more influenced by other factors such as inflammation which is suspected to be caused by CRF or hemodialysis procedures. This difference is also thought to be due to differences in the grouping of study subjects using BNP rather than ADMA. The sampling process was carried out before dialysis; hence, the post-dialysis reduction in ADMA and BNP levels and the possibility of other factors outside the kidney, namely DDAH overexpression, were poorly known. Jacobi et al. [28] in a meta-analysis study stated that ADMA levels in CKD patients increased 1.13-3.36 times as compared to controls. ADMA levels in CKD patients ranged from 0.46 µmol/l to 4.36 µmol/l, whereas the ADMA levels in HD patients ranged from 0.59 µmol/l to 6.0 µmol/l. There is no significant evidence of differences in the use of dialysis membranes or modalities of hemodialysis therapy in eliminating ADMA. Increasing the dose and frequency of hemodialysis is also said to be ineffective in reducing ADMA levels. Grooteman et al. [29] stated that it is better to control the factors associated with increased ADMA than using various methods of hemodialysis.

The proportion of fluid overload in this study was 64.3% with a median plasma BNP value of 663.75 (281.93-1666.28) pg/ml. The proportion of fluid overload in this study was higher than the study by Devolder et al. (22.3%) [30], Chazot et al. [16] (33.4%), and Zoccali et al. [31] (46%) in chronic hemodialysis patients three times weekly. A study by Chazot et al. [16] found plasma BNP >356 pg/ml referring to fluid overload conditions and having a high risk of death within six years in chronic hemodialysis patients three times weekly. The high plasma BNP in this study is thought to be related to less hemodialysis frequency; hence, there could be a potential for more interdialytic water accumulation.

This study found no significant relationship between endothelial dysfunction and fluid overload (RR: 1.042 p=0.832 95% CI: 0.714-1.521). High levels of BNP are not necessarily followed by increased levels of ADMA. Likewise, in the group with BNP levels ≤356 pg/ml, 48.9% have high levels of ADMA. This shows a tendency that increased ADMA levels are influenced by factors other than fluid overload such as age, inflammation, hypertension medications, DM, dyslipidemia, obesity, smoking, ultrafiltration, and CRF itself. Another factor that is thought to influence the results of this study is the time and frequency of sampling which is only done before hemodialysis. It is known that elevated ADMA levels occurred in CRF patients who are undergoing hemodialysis and those who are not undergoing hemodialysis due to traditional and non-traditional factors including CRF and hemodialysis procedures. Five hours after hemodialysis, ADMA will decrease by 65% and increase again 18 hours after hemodialysis. The decrease in ADMA levels varies greatly and is not necessarily followed by a decrease in BNP levels [27]. Chazot et al. [32] described a significant decrease in plasma BNP levels in the second and third months after undergoing chronic hemodialysis, but after the third month of undergoing chronic hemodialysis three times a week, plasma BNP levels did not differ significantly and formed a plateau pattern. This matter is suspected of causing no significant relationship between ADMA and BNP. Bentli et al. [24] stated that there was no significant correlation between ADMA and NT-proBNP (p>0.05). Additionally, there was a positive correlation between ADMA and apelin serum (r=0.343; p=0.007), and there was a negative correlation between apelin and NT-proBNP (r=0.303; p=0.019). Elevated levels of NT-proBNP and BNP are strong independent predictors of clinical events in heart failure patients and of maintaining left ventricle function. In the overexpression of natriuretic peptides in heart failure, the level of apelin is reduced. These factors possibly contribute to the decrease in apelinergic expression in heart failure, which includes myocardial stretch and activation of the renin-angiotensin and aldosterone systems. Shahawy et al. [33] stated that there was no correlation between ADMA and left ventricle myocardial infarction or ejection fraction percentage (p>0.05). Kandarini et al. [34] stated that large ultrafiltration had a role in reducing NO levels but did not play a clear role in reducing ADMA and ET-1 levels. On the contrary, the results of the study by Dogan et al. [35] stated that there was a correlation between ADMA with vena cava inferior (p<0.001) and carotid artery intima-media thickness (p=0.022). This difference is thought to be caused by the use of different hydration status parameters.

There was no relationship between endothelial dysfunction and fasting blood sugar control (95% CI 0.805 (0.512-1.265); p=0.440). After logistic regression was performed, it was found that fasting blood sugar control was not a confounding factor in the relationship between BNP and ADMA (OR (95% CI) 1.614 (0.551-4.728); p=0.383; ΔOR = 0.62% or <10%). This is in contrast with findings from Eliana et al. [36], which stated that there was a relationship between ADMA and blood sugar control (HbA1c) (p=0.01; r=0.720) and postprandial blood sugar (p=0.003; r=0.457) in 41 non-diabetic women CRF. Similarly, a study conducted by Dal et al. [37] stated that in the non-CKD DM population, a decrease in BNP levels was positively correlated with a decrease in HbA1c (r=0.345; p=0.003) and fasting blood sugar (r=0.366, p=0.002). Coutinho et al. [38] concluded that endothelial dysfunction often occurs in patients with CKD. However, DM is not involved in the occurrence of endothelial dysfunction, and uremia plays a major role. This study is in accordance with Coutino’s statement that blood sugar control is not a confounding factor in the relationship between BNP and ADMA, but uremia may play a more important role and inflammation is the main confounding factor.

There was no significant relationship between hypertension drugs and endothelial dysfunction (ADMA) (p=0.472) and was not a confounding factor for the relationship between fluid overload and endothelial dysfunction (OR (95% CI) 1.628 (0.553-4.790; p=0.376; ΔOR 0.85%). The results of previous studies stated that the calcium-channel blocker and angiotensin-receptor blocker indicated that there was a significant decrease in ADMA and BNP levels in the administration of these drugs, but the role of the two drug classes may be obscured by the inflammatory process that occurs in hemodialysis patients [25]. Weight status was not related to endothelial dysfunction (p=0.740) and was also not a confounding factor in the relationship between fluid overload and endothelial dysfunction (OR (95% CI) 1.622 (0.550-4.778)); p=0.381; ΔOR 0.37%). This may be due to the data grouping which only divides into two groups, namely obese and non-obese, which might oversimplify the relationship between variables. In Table 3, the two groups that describe the proportion between ADMA ≥100 ng/ml and ADMA <100 ng/ml seemed balanced. Hence, it was thought that there were other influencing factors including chronic inflammation. The relationship between age and fluid overload and endothelial dysfunction was not significant because perhaps the median sample in this study was relatively younger, namely 52 (47-62) years. Therefore, the risk of developing fluid overload was lower than that of the elderly. Nutritional status variables such as hypoalbumin cannot be analyzed because of the very large range of differences in proportions between the normal and hypoalbumin groups (Table 1).

After multivariate analysis and logistic regression, it was found that chronic inflammation was the main confounding variable affecting the relationship between fluid overload (BNP) and endothelial dysfunction (ADMA) (OR (95% CI) 1.604 (0.551-4.666) ΔOR 53.37%). Dogan et al. [35] stated that there was a relationship between high-sensitivity C-reactive protein (HsCRP) and ADMA (r=0.252; p<0.001). Additionally, Jacobs et al. [39] in their longitudinal study concluded that there was a significant relationship between fluid overload, BNP, and inflammation (HsCRP).

To the author’s knowledge, this is the first study that describes the relationship between fluid overload and endothelial dysfunction in twice-weekly hemodialysis patients. In addition, by knowing the levels of BNP, ADMA, and HsCRP biomarkers in hemodialysis patients, we are able to assess the patient's prognosis. Vallicova et al. stated that increased levels of ADMA, BNP, and HsCRP increased the risk of death from CVD [40]. Mallamaci et al, in a cohort study, stated that the combination of increasing the value of the three biomarkers increased mortality due to cardiovascular events by 10.5% and 11.6% for all causes of death (p≤0.01) [41]. However, this study also has limitations. This study is a cross-sectional study which causes researchers to be unable to control the factors that affect the variables studied in the long term. Furthermore, this study also did not examine BNP and ADMA levels after hemodialysis. Therefore, the effect of the hemodialysis procedure on these parameters cannot be observed.

## Conclusions

High levels of BNP are not necessarily followed by increased levels of ADMA; hence, there is no significant relationship between endothelial dysfunction and fluid overload. The most presumptive causes of CRF in this study were glomerulonephritis, DM, and hypertension. To prove the correctness of the theory of the relationship between fluid overload and endothelial dysfunction, it is necessary to conduct a study with a prospective cohort design that measures BNP and ADMA levels simultaneously after and before hemodialysis eight times.

## Appendices

Supplementary 1: informed consent form in Bahasa Indonesia

Penjelasan Mengikuti Penelitian

(Formulir Informed Consent)

Pada saat ini Divisi Ginjal Hipertensi Departemen Ilmu Penyakit Dalam FKUI/RSUPN Dr. Cipto Mangunkusumo (RSCM) mengadakan penelitian untuk mengetahui faktor-faktor klinis yang dapat memprediksi kadar BNP plasma lebih dari 356 pg/ml pada pasien HD dua kali seminggu. Bapak/Ibu dengan penyakit ginjal tahap akhir yang sudah menjalani HD dua kali seminggu diminta ikut serta dalam penelitian ini.

Pasien PGK di Indonesia terus meningkat dari tahun ke tahun. Sebagai konsekuensinya dibutuhkan tindakan HD kronik terus meningkat. Air dalam tubuh yang berlebih berakumulasi secara kronik menimbulkan kondisi hiperhidrasi, pada pasien HD kronik meningkatkan risiko kematian terutama terkait komplikasi penyakit jantung.

Keterbatasan frekuensi HD kronik saat ini di Indonesia umumnya dilakukan dua kali seminggu, lebih sedikit dari yang direkomendasikan atau yang umum dilakukan di luar negeri, sehingga kemungkinan lebih mudah terjadi penumpukan air dalam tubuh (hiperhidrasi) yang akan meningkatkan kadar BNP plasma. Salah satu metode pengukuran kelebihan air tubuh pada pasien HD kronik adalah dengan pengukuran kadar BNP plasma, selain menggambarkan kondisi air berlebih (hiperhidrasi), juga terkait dengan kondisi jantung (hipertrofi jantung kiri dan ganggguan fungsi sisitolik jantung), sesuai penelitian oleh Chazot dkk (2017), didapatkan pasien HD kronik dengan atau tanpa riwayat penyakit jantung, bila kadar BNP plasma >356 pg/ml, berisiko kematian 4,09 kali dalam periode waktu 6 tahun. Sehingga dirasa perlu dilakukan penelitian untuk mengetahui apakah usia, tekanan darah sistolik, interdialytic weigh gain (penambahan berat badan interdialsis), dan volume ultrafiltrasi dapat memprediksi kadar BNP plasma lebih dari 356 pg/ml pada pasien HD dua kali seminggu.

Selain dilakukan pemeriksaan status air didalam tubuh juga dilakukan pemeriksaan penanda disfungsi endotel/kerusakan sel endotel yang merupakan faktor risiko penyakit kardiovaskular dan faktor kematian akibat penyakit kardiovaskuler yaitu ADMA.

Apabila Bapak/Ibu berpartisipasi dalam penelitian, maka Bapak/Ibu akan diwawancarai dan dilakukan pemeriksaan kadar BNP dan ADMA plasma dengan mengambil sampel darah vena sekitar 5 ml.

Partisipasi Bapak/Ibu dalam penelitian ini tidak dipungut biaya dan semua informasi yang didapat dari penelitian ini dijaga kerahasiaannya. Keikutsertaan Bapak/Ibu bersifat sukarela dan tidak ada sanksi apapun bila Bapak/Ibu tidak bersedia ikut dengan alasan apa pun. Bapak/Ibu bebas untuk membatalkan keikutsertaannya pada penelitian ini kapan saja.

Apabila ada hal yang kurang jelas sehubungan dengan keikutsertaan Bapak/Ibu pada penelitian ini, dapat menghubungi peneliti:

Adi Wijaya, dr., SpPD

Divisi Ginjal Hipertensi, Departemen Ilmu Penyakit Dalam

RSUPN Dr. Cipto Mangunkusumo

Jl. Diponegoro 71, Jakarta Pusat

Hp/wa. 0813 812 652 99

Supplementary 2: subject sample size formula

$n=((z\alpha\sqrt{2P Q})+z\beta\sqrt{(P_1 Q_1+P_2 Q_2 )}/(P_1-P_2))^2= 62$

n: number of subjects in one group

zα: level of significance based on 95% confidence interval (α = 5%)

zβ: positive deviation based on 80% power (β = 20%)

Q: 1 - P

Q1: 1 - P1

Q2: 1 - P2

P1-P2: difference in proportion considerably significance (0.25)

P2: proportion in the group with no risk

P1: proportion in group with risk (62%)

Supplementary 3: blood sample size formula

 $n=(Z\alpha ^2 P (1-P))/(P.d^2) = 94$

n: number of samples needed

Zα: type I error. If α = 5%, then Zα = 1,960

P: prevalence (62%)

d: level of absolute accuracy (10 %)
